# Nomogram and Machine Learning Models Predict 1-Year Mortality Risk in Patients With Sepsis-Induced Cardiorenal Syndrome

**DOI:** 10.3389/fmed.2022.792238

**Published:** 2022-04-29

**Authors:** Yiguo Liu, Yingying Zhang, Xiaoqin Zhang, Xi Liu, Yanfang Zhou, Yun Jin, Chen Yu

**Affiliations:** Department of Nephrology, School of Medicine, Tongji Hospital, Tongji University, Shanghai, China

**Keywords:** sepsis, cardiorenal syndrome, prognosis, nomogram, machine learning

## Abstract

**Objective:**

Early prediction of long-term outcomes in patients with sepsis-induced cardiorenal syndrome (CRS) remains a great challenge in clinical practice. Herein, we aimed to construct a nomogram and machine learning model for predicting the 1-year mortality risk in patients with sepsis-induced CRS.

**Methods:**

This retrospective study enrolled 340 patients diagnosed with sepsis-induced CRS in Shanghai Tongji Hospital between January 2015 and May 2019, as a discovery cohort. Two predictive models, the nomogram and machine learning model, were used to predict 1-year mortality. The prognostic variables used to develop the nomogram were identified based on a forward stepwise binary logistic regression, and the predictive ability of the nomogram was evaluated by the areas under the receiver operating characteristic curve (AUC) and the calibration curve. Meanwhile, machine learning (ML) techniques, such as support vector machine, random forest (RF), and gradient boosted decision tree, were assessed mainly by accuracy and AUC. Feature ranking analysis was performed using the ML algorithm. Both nomogram and ML models were externally validated by an independent cohort of 103 patients diagnosed with sepsis-induced CRS between June 2019 and December 2020.

**Results:**

Age, sequential sepsis-related organ failure score (SOFA), serum myoglobin (MYO), vasopressor use, and mechanical ventilation were identified as independent risk factors for 1-year mortality in the nomogram predictive model. In the discovery cohort, the nomogram yielded higher AUC for predicting mortality than did the SOFA score (0.855 [95% CI: 0.815–0.895] vs. 0.756 [95% CI: 0.705–0.808]). For ML, the model developed by RF showed the highest accuracy (0.765) and AUC (0.854). In feature ranking analysis, factors such as age, MYO, SOFA score, vasopressor use, and baseline serum creatinine were identified as important features affecting 1-year prognosis. Moreover, the nomogram and RF model both performed well in external validation, with an AUC of 0.877 and 0.863, respectively.

**Conclusion:**

Our nomogram and ML models showed that age, SOFA score, serum MYO levels, and the use of vasopressors during hospitalization were the main factors influencing the risk of long-term mortality. Our models may serve as useful tools for assessing long-term prognosis in patients with sepsis-induced CRS.

## Introduction

Sepsis is a systemic inflammatory syndrome caused by the body’s unbalanced response to infection. Despite recent advances in diagnosis, treatment, and post-acute care, sepsis remains a deadly disease with unacceptably high morbidity and mortality rates (1). Sepsis can induce multiple organ dysfunction, including that of the heart and kidney, which may manifest clinically as myocardial depression, elevated myocardial injury markers, and acute renal insufficiency (2, 3). Cardiorenal syndrome (CRS) is an acute or chronic dysfunction of the heart or kidney that results in the acute or chronic dysfunction of the other organ. In 2008, the Acute Diseases Quality Initiative proposed five types of CRS, with the combined occurrence of heart and kidney dysfunction caused by sepsis and other systemic diseases defined as CRS type 5 (4). The mortality rates of patients with sepsis and CRS range from 20 to 60%. In addition, sepsis patients with newly diagnosed CRS are at a higher risk of developing a worse in-hospital prognosis than those without acute cardiorenal attack (5, 6). Despite the increasing attention being paid to this critical disease (sepsis-induced CRS), information about its prognostic factors and mortality rate remains limited to date.

The prediction of long-term outcomes in patients with sepsis-induced CRS may help to optimize decision-making and post-acute care. Machine learning (ML) techniques are currently being used as powerful and reliable tools for outcome assessment. Compared with standard methods of statistical model establishment, ML methods are capable of processing a larger number of variables and tend to output more accurate and precise results (7). A nomogram is an ancient calculator similar to the slide rule, (8) that provides graphical depictions of the logistic or Cox regression model. It has been used epidemiologically in disease diagnosis (9, 10), prognosis evaluation (11–13), and recurrence prediction (14). Although sophisticated ML methodologies may provide more accurate and generalized prediction models (15), the convenient and transparent characteristics of the nomogram still make it popular with clinicians.

Both traditional and ML models may serve as useful tools for improving prognostic judgment and decision-making by clinicians and for helping patients and their families understand the severity and prognosis of sepsis-induced CRS. Thus, this study aimed to explore the independent risk factors of 1-year mortality in sepsis-induced CRS patients and develop predictive models using traditional statistical methods and ML techniques.

## Materials and Methods

### Study Design and Population

This was a single-center, retrospective cohort study in which consecutive patients who developed concomitant acute cardiac and kidney injury secondary to sepsis during hospitalization at Shanghai Tongji Hospital from January 1, 2015, to December 31, 2020, were enrolled. The inclusion criteria were as follows: age ≥ 18 years; sepsis diagnosis in accordance with the third international consensus definitions published in 2016 (1); acute kidney injury (AKI) defined according to the 2012 Kidney Disease Improving Global Outcomes guidelines (16); acute myocardial injury indicated by at least of the following laboratory indicators: type B natriuretic peptide (BNP) increased ≥ 100 pg/mL, N-terminal pro-B-type natriuretic peptide (NT-proBNP) increased ≥ 300 pg/mL, and cardiac troponin I (cTnI) increased ≥ 0.03 ng/ml, within 48 h. The exclusion criteria were as follows: hospital length of stay ≤ 48 h; acute heart and kidney damage caused by non-infectious factors (e.g., urinary tract obstruction, autoimmune disease-related AKI, coronary heart disease complicated by acute myocardial infarction, etc.); active malignant tumors; mental disorders; pregnancy; and incomplete clinical data.

**FIGURE 1:**
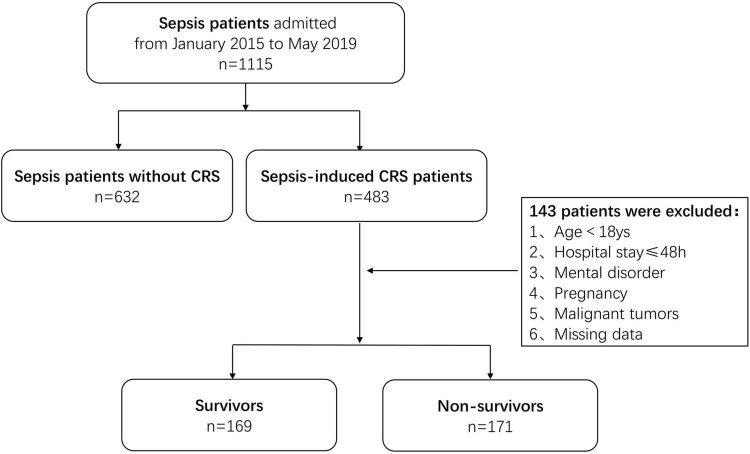
Flow chart illustrating the patient enrollment in discovery cohort.

In the discovery cohort, a total of 1,115 patients diagnosed with sepsis at Shanghai Tongji Hospital between January 2015 and May 2019 were screened, and 483 of them had concomitant acute cardiac and kidney injury secondary to sepsis during hospitalization. Of the 483 patients initially identified, 143 were excluded according to the above criteria. Finally, 340 patients with sepsis-induced CRS were enrolled and categorized according to their living status (alive or deceased) within 1 year after diagnosis as survivors (*n* = 169 patients) and non-survivors (*n* = 171 patients) (Figure 1). In the validation cohort, a total of 103 patients diagnosed with sepsis-induced CRS between June 2019 and December 2020 were enrolled by the same process (Supplementary Figure 1). To date, sepsis-induced CRS remains a clinical diagnosis characterized by simultaneous existence of acute heart and kidney injury in the setting of sepsis (17). Considering the lack of clear and consistent diagnostic criteria for sepsis-induced CRS, in our study, heart and kidney injury were evaluated using blood markers including BNP, NT-proBNP, cTnI, and serum creatinine (Scr) (18).

### Data Collection and Follow-Up

Data on patient characteristics, including age, sex, department, infection site, blood culture, vital signs, smoking history, pre-existing diseases, medication history, and in-hospital treatment, were retrieved from the medical records. Vital signs included blood pressure, heart rate, axillary temperature, and respiratory rate recorded in the nursing chart on the first day of diagnosis of sepsis-induced CRS. Medication history 3 months prior to diagnosis was reviewed and determined from both outpatient and inpatient records. In-hospital treatment comprised mechanical ventilation and the use of vasopressors such as dopamine, norepinephrine, or epinephrine.

Laboratory indices of cTnI, MYO, and Scr were recorded on the first and third days of diagnosis. Based on the linear analysis between the covariates and mortality risk, continuous variables such as cTnI and Scr were transformed into categorical variables. Additionally, to explore whether the rate of change in continuous variables would affect prognosis, we calculated rate of change using laboratory data from the first and third days of diagnosis. Rate of change in cTnI and baseline Scr were not considered in the logistic regression as some of the denominators of cTnI could be 0. For baseline Scr, we found a large multicollinearity between the creatinine level on the third day and the rate of change in baseline Scr.

The baseline Scr level was measured in accordance with the varying conditions of the patients in the following order: (1) the Scr value from the most recent examination before hospitalization (within 12 months); (2) the nadir creatinine value measured during the first 3 days of hospitalization; and (3) the baseline Scr value inversely deduced using the back-estimation formula according to the population’s average GFR level of 75 ml/(min⋅1.73 m^2^), (19). The SOFA scores were determined by the clinician from clinical records or from the medical records and laboratory values obtained on the first day. The patients were followed up for at least 1 year after discharge, and deaths were confirmed by either electronic medical records or telephone follow-ups.

### Statistical Analysis

#### Nomogram Prediction Model

**FIGURE 2:**
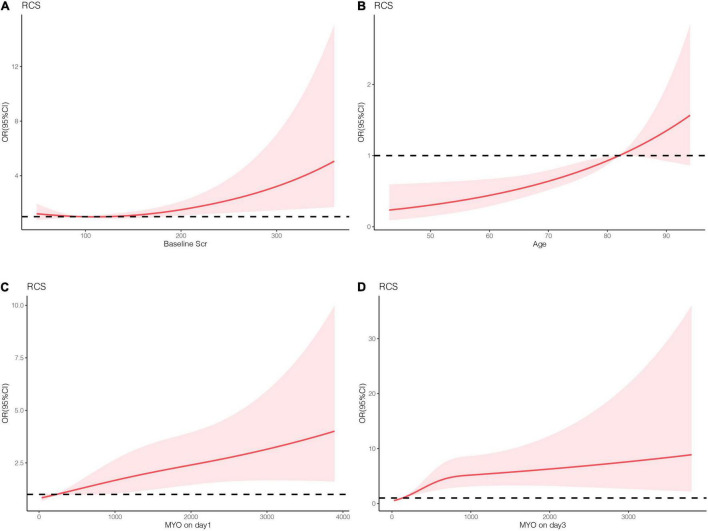
The linear analysis of numeric variables. **(A)** Basic serum creatinine. **(B)** Age. **(C)** Serum MYO levels on day 1. **(D)** Serum MYO levels on day 3.

**FIGURE 3:**
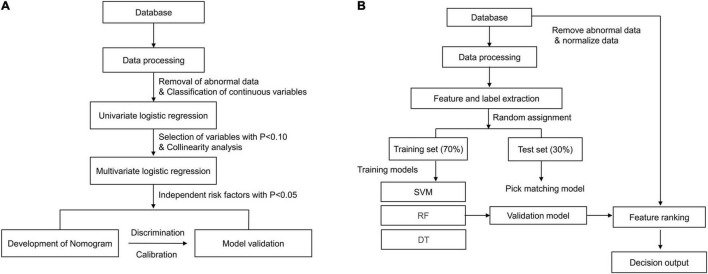
**(A)** The process of development of nomogram. **(B)** The process of machine learning methods.

Patient characteristics are presented as percentages for categorical data and as median with interquartile range for continuous data. As for traditional (non-ML) statistical methods, restricted cubic spine was used to assess the linear relationship between the potential variables and the outcome (Figure 2). In addition, non-linear factors were converted into categorical variables. Potential prognostic factors were evaluated using univariate logistic analysis, and the predictors (*p* < 0.10) were included in multiple logistic regression. The predictors enrolled in the nomogram met the requirement of 10 events per variable to reduce bias and variability of the logistic model. The variance inflation factor was used to test for multicollinearity between the predictors. Forward stepwise binary logistic regression was performed to identify the independent risk factors. The nomogram prediction model was then established based on independent prognostic factors, and internal and external validation were performed using the 500 bootstrap resampling method. The model’s discrimination ability was evaluated using the receiver operating characteristic curve (AUC), while the calibration ability was assessed using the calibration curve. The model accuracy was determined according to the Brier score, with a closer score to 0 indicating better accuracy. Statistical analyses were performed using IBM SPSS Statistics 22.0 and R software (R 4.0.2). Statistical significance was set at *p* < 0.05. The development process of the nomogram is shown in Figure 3A.

#### Machine Learning Model

The establishment and internal evaluation process of the ML model is shown in Figure 3B. The 1-year survival status was set as the label, and the other variables were set as features. Under fivefold cross-validation, 70% of the data were randomly assigned to the training set, and the remaining 30% were entered into the test set. In the training set, the predictive models (also called “classifiers”) were trained using ML algorithms such as support vector machine, random forest (RF), gradient boosted decision tree, extreme gradient boosting, and light gradient boosted machine. The classifiers were then applied to the test set for internal validation. Finally, the classifiers were externally verified in the validation cohort. The generalization capability of different classifiers was assessed by accuracy, recall, precision, F1 score, and AUC. Moreover, feature ranking analysis was performed, and those with a significant impact on survival were included in the decision tree algorithm. The developed and visualized decision tree was subjected to a post-pruning process to prevent overfitting of the data set. In the pruned decision tree, the 1-year mortality risks were classified as low (< 25%), moderate (25–49%), high (50–74%), and very high (> 75%) risk. The above algorithms were all run using Python software.

## Results

### Patient Characteristics

Of the 340 patients enrolled in the discovery cohort, 171 (50.3%) died within 1 year after the diagnosis of sepsis-induced CRS. The non-survivors were significantly older than the survivors (Z = –2.358, *P* = 0.018). Regarding infection, the most common was respiratory (56.8%), followed by digestive (19.1%), urinary tract (16.5%), and skin and soft tissue (5.0%) infections. Sex, vital signs, pre-existing diseases, and blood culture did not significantly affect prognosis. The baseline patient characteristics are shown in Table 1. In the validation cohort, 103 patients were enrolled, and 56 (54.4%) of them died within 1 year. The baseline Scr in the validation cohort was lower than that in the discovery cohort. Most of the baseline characteristics showed no statistical differences and were comparable in both cohorts (Supplementary Table 1).

**TABLE 1:** Patient clinical characteristics.

Variables	Survivors (*n* = 169)	Non-survivors (*n* = 171)	Z/χ^2^	*P*
Age	79.00 (64.50,86.00)	83.00 (72.00,88.00)	–2.358	0.018
Male sex	88 (52.1%)	97 (56.7%)	1.314	0.252
Department			10.181	0.017
ICU	58 (34.3%)	52 (30.4%)		
Emergency department	70 (41.4%)	55 (32.2%)		
Medical ward	27 (16.0%)	52 (30.4%)		
Surgical ward	14 (8.3%)	12 (7.0%)		
Infection site			9.708	0.021
Respiratory system	82 (48.5%)	111 (64.9%)		
Digestive system	37 (21.9%)	28 (16.4%)		
Urinary system	35 (20.7%)	21 (12.3%)		
Skin and soft tissue	8 (4.7%)	9 (5.3%)		
Other	7 (4.1%)	2 (1.2%)		
Blood culture			5.300	0.258
Negative	97 (57.4%)	81 (47.4%)		
Gram-positive	8 (4.7%)	15 (8.8%)		
Gram-negative	25 (14.8%)	29 (17.0%)		
Fungus	11 (6.5%)	9 (5.3%)		
Polymicrobial infection	28 (16.6%)	37 (21.6%)		
SBP, mmHg	120.00 (100.00, 140.00)	120.00 (105.00, 140.00)	–0.237	0.812
DBP, mmHg	70.00 (60.00, 80.00)	70.00 (60.00, 80.00)	–0.770	0.441
HR	85.00 (80.00, 99.00)	86.00 (80.00, 102.00)	–0.438	0.661
T, °C	37.00 (36.50, 38.00)	37.00 (36.50, 37.50)	–1.874	0.061
Pre-existing disease				
Diabetes	57 (33.7%)	54 (31.6%)	0.178	0.673
Hypertension	108 (63.9%)	108 (63.2%)	0.020	0.886
CAD	55 (32.5%)	59 (34.5%)	0.146	0.702
Stroke	51 (30.2%)	65 (38.0%)	2.321	0.128
CKD	25 (14.8%)	29 (17.0%)	0.299	0.585
History of tumor	16 (9.5%)	13 (7.6%)	0.379	0.538
History of smoking	28 (16.6%)	42 (24.6%)	3.322	0.068
Medication history				
Diuretics	62 (36.7%)	77 (45.0%)	2.448	0.118
CCB	53 (31.4%)	55 (32.2%)	0.026	0.874
ACEI	18 (10.7%)	10 (5.8%)	2.595	0.107
ARB	48 (28.4%)	44 (25.7%)	0.307	0.579
β-blocker	41 (24.3%)	34 (19.9%)	0.947	0.330
Statin	45 (26.6%)	37 (21.6%)	1.156	0.282
Nitrate ester	36 (21.3%)	35 (20.5%)	0.036	0.850
Digoxin	14 (8.3%)	20 (11.7%)	1.099	0.297
Antiplatelet drug	59 (34.9%)	52 (30.4%)	0.783	0.376
Warfarin	37 (21.9%)	27 (15.8%)	2.073	0.150
In-hospital treatment				
Mechanical ventilation	24 (14.2%)	68 (39.8%)	28.146	<0.001
Vasopressor	63 (37.3%)	126 (73.7%)	45.632	<0.001
qSOFA				
≤2	123 (72.8%)	119 (69.6%)	0.422	0.516
>2	46 (27.2%)	52 (30.4%)		
Total SOFA	5.00 (3.00, 8.00)	11.00 (6.00, 13.00)	–8.166	<0.001
Respiratory system	0.00 (0.00,2.00)	0.00 (0.00,3.00)	–5.755	<0.001
Nervous system	0.00 (0.00,1.00)	1.00 (1.00,3.00)	–8.921	<0.001
Cardiovascular system	0.00 (0.00,1.00)	2.00 (0.00,3.00)	–5.917	<0.001
Liver	0.00 (0.00,1.00)	0.00 (0.00,1.00)	–2.049	0.040
Coagulation	1.00 (1.00,2.00)	1.00 (1.00,2.00)	–1.027	0.305
Kidneys	2.00 (1.00,2.00)	2.00 (1.00,3.00)	–2.857	0.004
Laboratory variables				
Baseline Scr, umol/L	93.00 (72.00, 130.50)	98.00 (74.00, 162.00)	–1.574	0.116
Scr on day 1, umol/L	185.00 (141.00, 260.50)	189.00 (146.00, 289.00)	–0.753	0.452
Scr on day 3, umol/L			3.600	0.308
<133	73 (43.2%)	61 (35.7%)		
133∼177	33 (19.5%)	30 (17.5%)		
178∼442	50 (29.6%)	60 (35.1%)		
>443	13 (7.7%)	20 (11.7%)		
MYOon day 1, ng/mL	174.20 (81.60,484.80)	316.10 (126.60,1055.40)	–3.854	<0.001
MYO on day 3, ng/mL	94.10 (49.30,168.90)	226.90 (90.60,650.00)	–6.709	<0.001
The rate of change in MYO, %	–49.00 (-73.00, 0.00)	–28.00 (-66.00,24.00)	–2.932	0.003
cTnI on day 1, ng/mL			7.441	0.024
< 0.03	15 (8.9%)	22 (12.9%)		
0.03∼0.5	105 (62.1%)	120 (70.2%)		
>0.5	49 (29.0%)	29 (17.0%)		
cTnI on day 3, ng/mL			0.329	0.848
<0.03	27 (6.0%)	31 (18.1%)		
0.03∼0.5	110 (65.1%)	110 (64.3%)		
>0.5	32 (18.9%)	30 (17.5%)		

*ICU, intensive care unit; SBP, systolic blood pressure; DBP, diastolic blood pressure; HR, heart rate; T, temperature; CAD, coronary artery disease; CKD, chronic kidney disease; CCB, calcium channel blocker; ACEI, angiotensin converting enzyme inhibitors; ARB, angiotensin receptor blockers; Serum creatinine, Scr; SOFA, Sequential (Sepsis-related) Organ Failure Assessment; qSOFA, quick SOFA; MYO, myoglobin; cTnI, cardiac troponin I.*

### Nomogram: Development and Assessment

**FIGURE 4:**
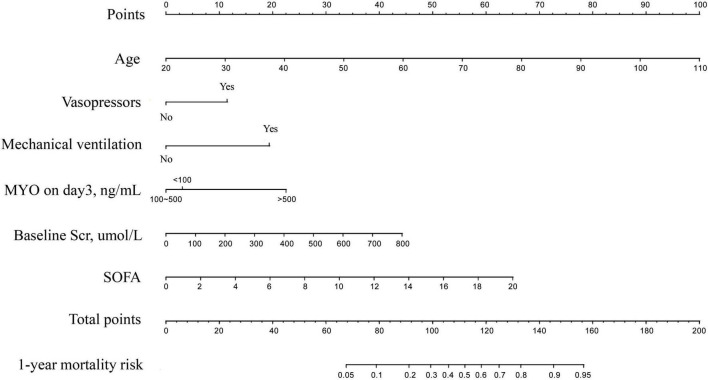
The Nomogram for prediction of 1-year mortality risk in patients with newly diagnosed sepsis-induced cardiorenal syndrome.

The results of univariate and multivariate analyses for risk factors of 1 year mortality are shown in Table 2. Age, SOFA, and serum MYO on day 3 of diagnosis, use of vasopressors, and mechanical ventilation were identified as independent risk factors for 1-year mortality. The final model was constructed based on the prognostic predictors and further visualized as a nomogram using the “rms” package from the R software (Figure 4). The application methods for the nomogram are described as follows. First, we drew an ascending line from the variable axis to the “Points” axis to obtain the points for each risk factor. Then, the scores of all the variables were combined to obtain the total number of points. Finally, we drew a downward perpendicular line from the “Total Points” axis to the “Risk” axis. The corresponding number was then presented as the estimated risk of 1-year mortality. The β-coefficients for the final logistic regression model are presented in Table 3.

**TABLE 2:** Univariate and multivariate analyses for prognostic factors.

Variables	Category	Univariate analysis		Multivariate analysis	
			
		OR (95% CI)	*p*-value	OR (95% CI)	*p*-value
Age	Per year	1.04 (1.02∼1.06)	0.000	1.08 (1.05∼1.11)	<0.001
Gender	Female	1			
	Male	1.21 (0.79∼1.85)	0.389		
SBP	Per mmHg	1.01 (0.99∼1.02)	0.103		
DBP	Per mmHg	1.00 (0.99∼1.02)	0.550		
HR	Per minute	1.00 (0.99∼1.01)	0.706		
T, Celsius	Per degrees Celsius	0.96 (0.87∼1.07)	0.510		
Infection site	Respiratory system	1	0.022	1	0.991
	Digestive system	0.56 (0.32,0.99)	0.045	1.15 (0.53∼2.47)	0.726
	Urinary system	0.44 (0.24,0.82)	0.009	0.92 (0.41∼2.06)	0.839
	Skin and soft tissue	0.83 (0.31,2.25)	0.715	0.88 (0.22∼3.49)	0.856
	Other	0.21 (0.04,1.04)	0.056	1.16 (0.16∼8.08)	0.878
Basic disease					
Diabetes	No	1			
	Yes	0.91 (0.58∼1.43)	0.673		
Hypertension	No	1			
	Yes	0.97 (0.62∼1.51)	0.886		
CAD	No	1			
	Yes	1.09 (0.70∼1.71)	0.702		
Stroke	No	1			
	Yes	1.419 (0.90∼2.23)	0.128		
CKD	No	1			
	Yes	1.17 (0.66∼2.11)	0.585		
Diabetes	No	1			
	Yes	0.79 (0.37∼1.69)	0.539		
History of smoking	No	1		1	
	Yes	1.64 (0.96∼2.80)	0.070	1.21 (0.59∼2.51)	0.592
Medication history	No				
Diuretics	Yes	1			
	No	1.41 (0.92∼2.18)	0.118		
CCB	No	1			
	Yes	1.04 (0.66∼1.64)	0.874		
ACEI	No	1			
	Yes	0.52 (0.23∼1.17)	0.112		
ARB	No	1			
	Yes	0.873 (0.54∼1.41)	0.579		
β-blocker	No	1			
	Yes	0.78 (0.46∼1.30)	0.331		
Statin	No	1			
	Yes	0.76 (0.46∼1.25)	0.283		
Nitrate ester	No	1			
	Yes	0.95 (0.56∼1.60)	0.850		
Digoxin	No	1			
	Yes	1.47 (0.72∼3.01)	0.297		
Antiplatelet drug	No	1			
	Yes	0.82 (0.52∼1.28)	0.376		
Warfarin	No	1			
	Yes	0.67 (0.39∼1.16)	0.151		
In-hospital treatment					
Mechanical ventilation	No	1		1	
	Yes	3.98 (2.35,6.77)	<0.001	3.51 (1.67∼7.40)	0.001
Vasopressor	No	1		1	
	Yes	4.71 (2.97∼7.48)	<0.001	2.07 (1.12∼3.83)	0.020
qSOFA	≤ 2	1			
	>2	1.17 (0.73∼1.87)	0.422		
Total SOFA		1.29 (1.21∼1.37)	<0.001	1.24 (1.14∼1.37)	<0.001
Laboratory variables					
Baseline Scr, μmol/L		1.00 (1.00∼1.01)	0.005	1.00 (0.99∼1.01)	0.108
Scr on day 1, μmol/L		1.00 (1.00∼1.00)	0.118	1.00 (0.99∼1.00)	0.763
Scr on day 3, μmol/L	< 133	1	0.311	1	
	133∼177	1.09 (0.60∼1.98)	0.783	0.61 (0.29∼1.52)	0.297
	178∼442	1.44 (0.87∼2.38)	0.161	1.68 (0.83∼3.46)	0.152
	> 443	1.84 (0.85∼4.00)	0.123	2.08 (0.72∼6.18)	0.182
MYO on day 1, ng/mL		1.00 (1.00∼1.00)	<0.001	1.00 (0.99∼1.00)	0.867
MYO on day 3, ng/mL	< 100	1	<0.001	1	0.036
	100∼500	2.23 (1.36∼3.64)	0.001	0.83 (0.41∼1.65)	0.588
	> 500	8.50 (4.19∼17.24)	<0.001	3.51 (0.98∼12.55)	0.054
The rate of change in MYO, %		1.00(1.00∼1.01)	0.012	1.61 (1.26∼2.14)	0.502
cTnI on day 1, ng/mL	< 0.03	1	0.026	0.81 (0.34∼1.95)	0.056
	0.03∼0.5	0.78 (0.38∼1.58)	0.489	0.36 (0.13∼1.00)	0.640
	> 0.5	0.40 (0.18∼0.90)	0.026	1.00 (0.13∼1.00)	0.050
cTnI on day 3, ng/mL	< 0.03	1	0.849		
	0.03∼0.5	0.87 (0.50∼1.56)	0.640		
	> 0.5	0.82 (0.40∼1.67)	0.580		

*SBP, systolic blood pressure; DBP, diastolic blood pressure; HR, heart rate; T, temperature; CAD, coronary artery disease; CKD, chronic kidney disease; CCB, calcium channel blocker; ACEI, angiotensin converting enzyme inhibitors; ARB, angiotensin receptor blockers; Serum creatinine, Scr; SOFA, Sequential (Sepsis-related) Organ Failure Assessment; qSOFA, quick SOFA; MYO, myoglobin; cTnI, cardiac troponin I.*

**TABLE 3:** Regression coefficient estimates of the 1-year predictive model.

Variables	β	SE	*P*	OR	95% CI
Age, year	0.073	0.013	<0.001	1.076	1.048∼1.105
Total SOFA	0.215	0.044	<0.001	1.240	1.137∼1.352
Vasopressors	0.758	0.299	0.011	2.133	1.188∼3.831
Mechanical ventilation	1.280	0.365	<0.001	3.598	1.760∼7.353
Baseline Scr, umol/L	0.004	0.002	0.066	1.004	1.001∼1.008
MYO on day 3, ng/mL					
<100	–	–	0.002	–	–
100∼500	–0.203	0.325	0.532	0.816	0.432∼1.542
>500	1.286	0.444	0.004	3.618	1.517∼8.629
Constant	–8.680	1.256	0.000	0.000	–

*SOFA, Sequential (Sepsis-related) Organ Failure Assessment; Scr, Scr; MYO, myoglobin.*

**FIGURE 5:**
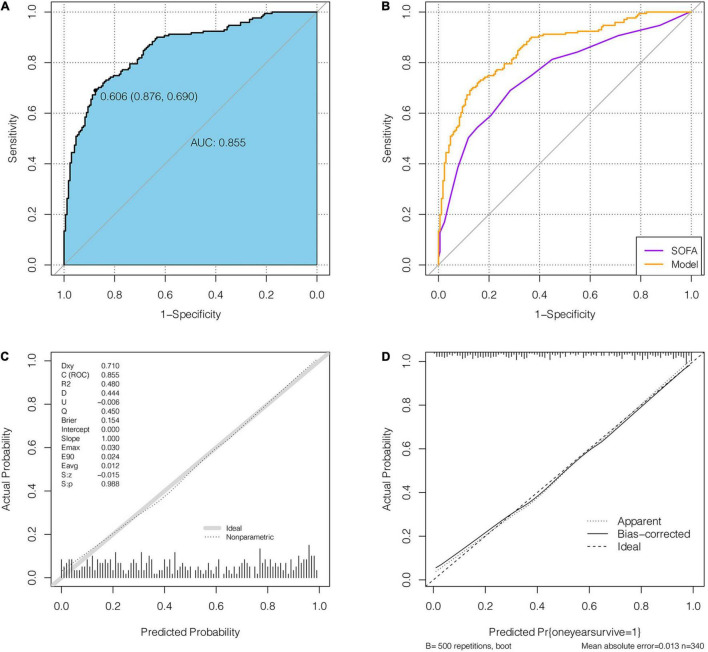
The receiver operating characteristic (ROC) curve and calibration curve of nomogram. **(A)** the ROC curves of the nomogram in discovery cohort. **(B)** Comparison of AUC between the nomogram and SOFA. **(C)** The calibration curve of the nomogram. **(D)** Comparison of the calibration curves between the ideal model, the nomogram and the bias-corrected model.

**FIGURE 6:**
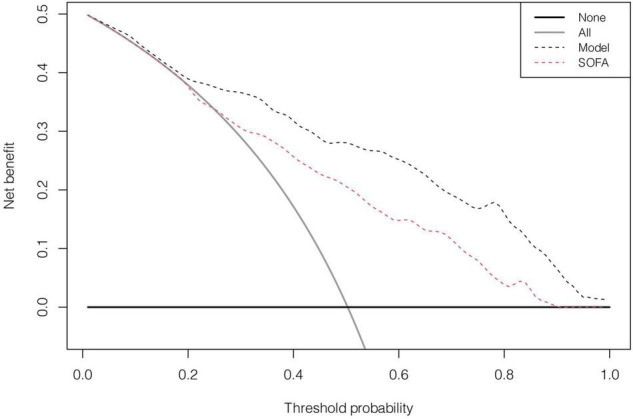
The decision analysis curves of the nomogram and SOFA in discovery cohort.

In the discovery cohort, the prognostic nomogram achieved excellent discrimination, with an AUC of 0.855 [95% confidence interval (CI): 0.815–0.895] (Figure 5A), and relatively low optimism, with a bootstrap validated AUC of 0.843. When SOFA score was solely used to predict 1-year outcome, it only achieved an AUC of 0.756 (95% CI: 0.705–0.808). DeLong test to compare the AUC between the SOFA score and nomogram showed that the nomogram had a significantly higher discrimination capability (Figure 5B). With respect to calibration capability, the calibration curve indicated a close consistency between the predicted and actual risks (Figure 5C). In internal validation, the nomogram predictive model demonstrated good concordance with the bootstrapped corrected model (Figure 5D). Further, it showed reasonably acceptable accuracy, with a Brier score of 0.154. In addition, decision curve analysis revealed that the nomogram yielded higher net benefits in predicting 1-year mortality than did the SOFA score (Figure 6). In external validation, the nomogram demonstrated strong discrimination, accuracy, and calibration power, as it achieved an AUC of 0.877 (95% CI: 0.812–0.941), a bias-corrected AUC of 0.844, a Brier score of 0.144, a bias-corrected Brier score of 0.171, and well-fitted calibration curves (Supplementary Figure 2). Moreover, Delong test and the decision curve analysis showed that the nomogram had better prognostic ability than the SOFA score in external validation (Supplementary Figure 3).

### Machine Learning Models: Selection and Evaluation

**FIGURE 7:**
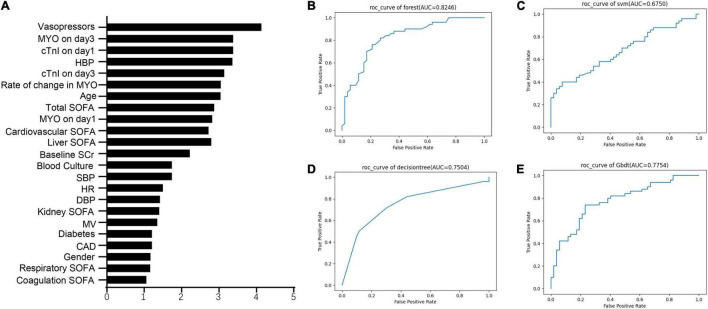
The feature importance and the ROC curves of machine learning models. **(A)** The feature ranking analysis produced by Random Forest. **(B)** ROC curves of Random Forest. **(C)** ROC curves of support vector machine (SVM). **(D)** ROC curves of decision tree. **(E)** ROC curves of gradient boosted decision tree (GBDT).

**FIGURE 8:**
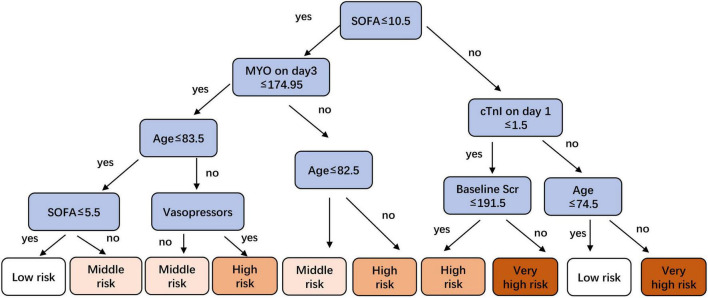
A pruned decision tree for predicting risk stratification of 1-year mortality in sepsis-induced CRS patients.

The metrics for generalization capability, such as accuracy and precision, for the various ML models are presented in Table 4, while the AUCs are shown in Figure 7. The RF model showed the highest accuracy and AUC at 0.765 and 0.854, respectively. The results of feature ranking are displayed in Figure 6A. Vasopressor use, serum MYO level on day 3 of diagnosis, rate of change in serum MYO, serum cTnI level on days 1 and 3, history of hypertension, and the SOFA score were important influencing factors of 1-year survival. After inputting the features of interest to the decision tree algorithm and post-pruning, the final decision tree was obtained (Figure 8). Based on this RF model, patients aged > 74.5 years with SOFA scores > 10.5 points and cTnI > 1.5 ng/mL on the first day of diagnosis, had a very high risk (> 75%) of 1-year mortality. In external validation, the RF model also showed good predictive ability by achieving an AUC of 0.863, a precision of 0.775 and a recall of 0.821 (Supplementary Figure 4).

**TABLE 4:** The metrics of different machine learning models.

Machine learning techniques	Accuracy	Precision	Recall	F1 score	AUC
Decision tree	0.657	0.627	0.740	0.679	0.750
SVM	0.637	0.741	0.840	0.778	0.675
Random forest	0.765	0.724	0.696	0.753	0.825
GBDT	0.716	0.691	0.760	0.724	0.775
Xgboost	0.706	0.667	0.800	0.727	0.708
lGBM	0.716	0.684	0.780	0.729	0.797

*SVM, support vector machine; GBDT, gradient boosted decision tree; Xgboost, extreme gradient boosting; LGBM, light gradient boosted machine.*

## Discussion

The prognostic factors of sepsis-induced CRS are yet to be established. We utilized blood tests which were routinely collected in clinical setting and determined the following five important risk factors that composed the predictive nomogram: age, SOFA, serum MYO levels, use of vasopressors, and mechanical ventilation during hospitalization. We also found that age, SOFA score, serum MYO levels, and vasopressor use were important predictors for the 1-year prognosis in ML models. The nomogram and ML models developed in our study showed good predictive ability and might help clinicians evaluate sepsis severity and predict long-term outcomes in the early diagnostic period.

Sepsis is a critical acute illness that is frequently associated with multiple organ dysfunction and high mortality rates. Thus, timely prediction of the long-term prognosis in patients with sepsis-induced CRS is of great importance in clinic therapy and post-acute care. The SOFA score is known to play a significant role in the diagnosis and in-hospital prognosis of sepsis patients (1, 20). However, even though SOFA includes assessment of the heart and kidney, the SOFA score alone cannot adequately predict the long-term prognosis of patients with sepsis-induced acute CRS. The current study identified five independent risk factors that strongly affect the 1-year prognosis. The nomogram comprising of these five factors along with baseline Scr showed significantly higher discrimination power and yielded greater net benefits in predicting 1-year outcomes in comparison to the model which used the SOFA score alone. Interestingly, we found serum MYO to be an independent risk factor in both multiple logistic regression and ML algorithms. MYO is a heme protein expressed in cardiomyocytes and skeletal muscle cells (21). Serum MYO levels are elevated in patients with acute myocardial infarction, renal insufficiency, muscle trauma, shock, and severe infection (22, 23). Recent studies have shown that serum MYO is strongly correlated with the severity of sepsis, and an increase in the serum MYO levels within 28 days after diagnosis indicates a higher risk for mortality (24). Yang et al. also found MYO to be an independent prognostic factor for septic shock (25). In sepsis-induced CRS, MYO is released from impaired cardiomyocytes and accumulates in the blood stream. It then deposits in the kidney tissue, further exacerbating kidney injury, and causing oxidative damage and lipid peroxidation (26). Our study showed that serum MYO levels > 500 ng/ml on the third day of diagnosis plays an important role in promoting long-term morbidity in patients with sepsis-induced CRS. To the best of our knowledge, our study is the first to report that the serum MYO level is an independent predictor of 1-year mortality in patients with sepsis-induced CRS.

Another interesting finding is that the higher demand for vasopressors or mechanical ventilation may have a profound impact on long-term health loss and therefore result in poorer outcomes. In 2001, Rivers et al. (27) recommended early goal-directed therapy (EGDT) that included the early use of vasopressors for patients with septic shock. However, subsequent research showed no difference in 90-day mortality rates between EGDT and usual care (28). Therefore, the effect of vasopressors on clinical outcomes remains controversial despite their rapid and significant effect on blood pressure. As for mechanical ventilation, previous studies have shown that activation of TLR4 in sepsis can induce increased expression of integrin β5, which can cause higher susceptibility to mechanical ventilation-related acute lung injury (29). Other studies have also suggested that sepsis patients are at risk of diaphragm contraction dysfunction after mechanical ventilation, which could lead to a higher possibility of weaning failure (30, 31). Similarly, our study found a correlation between mechanical ventilation and a worsening 1-year mortality, which may be associated with mechanical ventilation-related lung injury that could result in long-term organ dysfunction and decline in quality of life.

Sepsis patients with pre-existing kidney injury have a higher risk of developing AKI and multi-organ failure (32). However, there are conflicting reports on the outcomes of AKI or acute renal failure in patients with CKD. Several studies have reported higher mortality rates in acute renal failure patients with lower Scr levels (33, 34). Neyra et al. also reported that AKI (stage ≥ 2) in CKD is strongly associated with worse outcomes in patients with severe sepsis (35). However, our findings suggest that neither baseline renal function nor sepsis-induced AKI greatly influenced 1-year mortality. Treatment for AKI or CKD, such as renal replacement therapy, may have a positive effect on lowering the health impact of sepsis-induced AKI or CKD.

Regarding ML, we found that the classifier trained by the RF algorithm yielded the optimal predictive capability. The risk factors identified in the logistic regression model (e.g., age, vasopressor, MYO, and SOFA score) also ranked high in the feature analysis. However, we observed a few differences between the results of logistic regression and those of ML algorithm. First, cTnI was transformed and treated as a categorical variable throughout the logistic regression analysis owing to the non-linear relationship between cTnI and 1-year mortality. The results showed that cTnI did not influence 1-year mortality. In the ML algorithms, cTnI and MYO were all treated as numeric variables, and the results presented cTnI as an important prognostic factor. Second, given the limited sample size, only a few variables were included in the logistic regression model to prevent overfitting, and the total rather than the individual SOFA score per organ was included in the analysis. Under the same sample size, more variables were included in the ML models. The results showed that in addition to the total SOFA score, the cardiovascular and liver SOFA scores also had a significant effect on 1-year survival. Third, unlike the nomogram from the logistic regression model, the decision tree presented us with a flowchart of prognosis judgment in a risk-stratified manner. Although the decision tree had lower predictive capability for mortality than the nomogram, it had the advantage of including fewer variables in decision making, thus making it more efficient in risk judgment. Finally, in our study, the results of external validation indicated that RF model does not show a significantly higher predictive ability than the nomogram, which may be due to the limited sample size and predictor variables included in this study. We believe that this study serves as a preliminary exploration of ML models to predict mortality in sepsis-induced CRS, and a large-scale, multi-center clinical research should be conducted in the future to further improve and update the ML models.

Our study has some limitations. First, the development and external validation of the models were both performed in the dataset collected from Shanghai Tongji Hospital. Hence, validation of these models in different institutions is required before clinical application. Second, we recognize that apart from the predictors analyzed in this study, factors such as antibiotic use and left ventricular ejection fraction may also affect the outcome. Antibiotics were practically used in every patient with sepsis-induced CRS, but the type, dosage, and timing of the antibiotics varied greatly in the different patients, thus making it difficult to use a unified standard for data collection in this retrospective study. We also noticed that changes in left ventricular ejection fraction often reflected the severity of acute myocardial dysfunction. However, in the process of data collection, we found that the detection rate with echocardiography was apparently low, which may be due to the inconvenience associated with critically ill patients leaving the ward and the lack of utilization of bedside echocardiography outside the ICU and cardiology ward. In addition, the limited sample size has constrained the predictors included in the study. Therefore, we believe that more potential predictors should be included in the prospective study under a larger sample size. Third, new biomarkers for AKI, such as neutrophil gelatinase-related lipocalin and renal injury molecule 1, were not considered in this study as they were not tested in Shanghai Tongji Hospital during the study period. Future studies should include more sensitive biomarkers for model development.

## Conclusion

In conclusion, we constructed and internally validated predictive nomogram and ML models for the prediction of poor 1-year outcomes in patients with sepsis-induced CRS. The predictive models were developed by utilizing objective data routinely collected in clinical practice. Six optimal factors for predicting the 1-year prognosis were used to develop the clinical nomogram; they include age, SOFA score, mechanical ventilation, vasopressor use, baseline Scr, and serum MYO levels. Using both multiple logistic regression and ML algorithms, we found that age, SOFA score, vasopressor use, and serum MYO levels were key prognostic factors. In our study, both models showed strengths in predicting poor 1-year outcomes, and we believe this combination might be more clinically useful than the nomogram or ML model alone. Early prediction of long-term prognosis in patients with sepsis-induced CRS is of great importance and may greatly influence clinical treatment and post-acute care.

## Data Availability Statement

The original contributions presented in the study are included in the article/Supplementary Material, further inquiries can be directed to the corresponding author/s.

## Ethics Statement

The studies involving human participants were reviewed and approved by the ethics committee of Shanghai Tongji Hospital. Written informed consent for participation was not required for this study in accordance with the national legislation and the institutional requirements.

## Author Contributions

CY, YL, and YyZ contributed to conception and design of the study. YL collected the clinical data, organized the database, and wrote the first draft of the manuscript. YL and YyZ performed the statistical analysis. YyZ, XZ, YfZ, YJ, and CY revised the manuscript. All authors have contributed to the article and approved the submitted version.

## Conflict of Interest

The authors declare that the research was conducted in the absence of any commercial or financial relationships that could be construed as a potential conflict of interest.

## Publisher’s Note

All claims expressed in this article are solely those of the authors and do not necessarily represent those of their affiliated organizations, or those of the publisher, the editors and the reviewers. Any product that may be evaluated in this article, or claim that may be made by its manufacturer, is not guaranteed or endorsed by the publisher.

## Supplementary Material

Supplementary Figure 1 Flow chart illustrating the patient enrollment in validation cohort.

Supplementary Figure 2 The receiver operating characteristic (ROC) curve and calibration curve of nomogram in external validation. **(A)** The ROC curves of the nomogram; **(B)** comparison of AUC between the nomogram and SOFA; **(C)** the calibration curve of the nomogram; **(D)** comparison of the calibration curves between the ideal model, the nomogram and the bias-corrected model.

Supplementary Figure 3 The decision analysis curves of the nomogram and SOFA in external validation.

Supplementary Figure 4 The ROC curves of machine learning models in external validation. **(A)** ROC curves of Random Forest; **(B)** ROC curves of decision tree.

## Abbreviations

CRS: cardiorenal syndrome
AUC: area under the curve
ML: machine learning
SOFA: Sequential Organ Failure Assessment
MYO: myoglobin
Scr: serum creatinine
AKI: acute kidney injury
BNP: type B natriuretic peptide
cTnI: cardiac troponin I
NT-proBNP: N-terminal pro-B-type natriuretic peptide
RF: random forest.
