# Supplementary material for: Nomogram and Machine Learning Models Predict 1-Year Mortality Risk in Patients With Sepsis-Induced Cardiorenal Syndrome
Source: Front Med (Lausanne). 2022 Apr 29;9:792238. doi: 10.3389/fmed.2022.792238 (PMC9099150; doi:10.3389/fmed.2022.792238)
Supplement: Supplementary file 1 [file Table_1.DOCX]

**Supplementary Table 1.** Comparison of baseline characteristics between discovery cohort and validation cohort

| Variables | Discovery cohort  n=340 | Validation cohort  n=103 | Z/χ^2^ | *P* |
| --- | --- | --- | --- | --- |
| age | 82.00 (69.00, 87.00) | 81.00 (68.00, 87.00) | -0.019 | 0.985 |
| Male | 185 (54.4%) | 57 (55.3%) | 0.027 | 0.868 |
| department |  |  | 4.039 | 0.257 |
| ICU | 110 (32.4%) | 23 (22.3%) |  |  |
| Emergency department | 125 (36.8%) | 41 (39.8%) |  |  |
| Medical ward | 79 (23.2%) | 29 (28.2%) |  |  |
| Surgical ward | 26 (7.6%) | 10 (9.7%) |  |  |
| Infection site |  |  | 4.774 | 0.331 |
| Respiratory system | 193 (56.8%) | 51 (49.5%) |  |  |
| Digestive system | 65 (19.1%) | 21 (20.4%) |  |  |
| Urinary system | 56 (16.5%) | 19 (18.4%) |  |  |
| Skin and soft tissue | 17 (5.0%) | 5 (4.9%) |  |  |
| Other | 9 (2.6%) | 7 (6.8%) |  |  |
| Pre-existing disease |  |  |  |  |
| Diabetes | 111 (32.6%) | 31 (30.1%) | 0.236 | 0.627 |
| Hypertension | 216 (63.5%) | 55 (53.4%) | 3.416 | 0.065 |
| CAD | 114 (33.5%) | 27 (26.2%) | 1.950 | 0.163 |
| Stroke | 116 (34.1%) | 27 (26.2%) | 2.259 | 0.133 |
| CKD | 54 (15.9%) | 14 (13.6%) | 0.319 | 0.572 |
| History of tumor | 29 (8.5%) | 8 (7.8%) | 0.060 | 0.806 |
| In-hospital treatment |  |  |  |  |
| Mechanical ventilation | 92 (27.1%) | 28 (27.2%) | 0.001 | 0.980 |
| Vasopressor | 189 (55.6%) | 55 (53.4%) | 0.153 | 0.695 |
| qSOFA |  |  | 0.817 | 0.366 |
| ≤2 | 242 (71.2%) | 78 (75.7%) |  |  |
| >2 | 98 (28.8%) | 25 (24.3%) |  |  |
| Total SOFA | 7.00 (4.00, 11.00) | 8.00 (5.00, 12.00) | -1.230 | 0.219 |
| Respiratory System | 1.50 (0.00, 3.00) | 1.00 (1.00, 4.00) | -2.744 | 0.006 |
| Nervous System | 1.00 (0.00, 2.00) | 1.00 (0.00, 2.00) | -0.289 | 0.773 |
| Cardiovascular system | 0.00 (0.00, 3.00) | 1.00 (0.00, 4.00) | -3.540 | 0.001 |
| Liver | 0.00 (0.00, 1.00) | 0.00 (0.00, 1.00) | -0.339 | 0.735 |
| Coagulation | 1.00 (0.00, 2.00) | 2.00 (1.00, 2.00) | -2.328 | 0.020 |
| Kidneys | 2.00 (1.00, 3.00) | 1.00 (1.00, 2.00) | -4.110 | 0.001 |
| Laboratory variables |  |  |  |  |
| Baseline Scr, umol/L | 95.50 (73.25, 144.75) | 74.00 (53.00,98.00) | -4.872 | 0.000 |
| MYO on day 1,ng/mL | 228.75 (104.10, 710.35) | 190.90 (84.60,458.50) | -1.882 | 0.060 |
| MYO on day 3, ng/mL | 133.05 (64.8, 360.40) | 120.20 (59.60,416.70) | -0.379 | 0.705 |
| The rate of change in MYO, % | -43.5 (-70.00, 6.50) | -36.37 (-64.47-21.08) | -1.367 | 0.172 |

Abbreviation: ICU,intensive care unit; CAD, coronary artery disease; CKD, chronic kidney disease; Scr, serum creatinine; SOFA, Sequential (Sepsis-related) Organ Failure Assessment; qSOFA, quick SOFA; MYO, myoglobin.
