# Supplementary figures and images for: Nomogram and Machine Learning Models Predict 1-Year Mortality Risk in Patients With Sepsis-Induced Cardiorenal Syndrome
Source: Front Med (Lausanne). 2022 Apr 29;9:792238. doi: 10.3389/fmed.2022.792238 (PMC9099150; doi:10.3389/fmed.2022.792238)

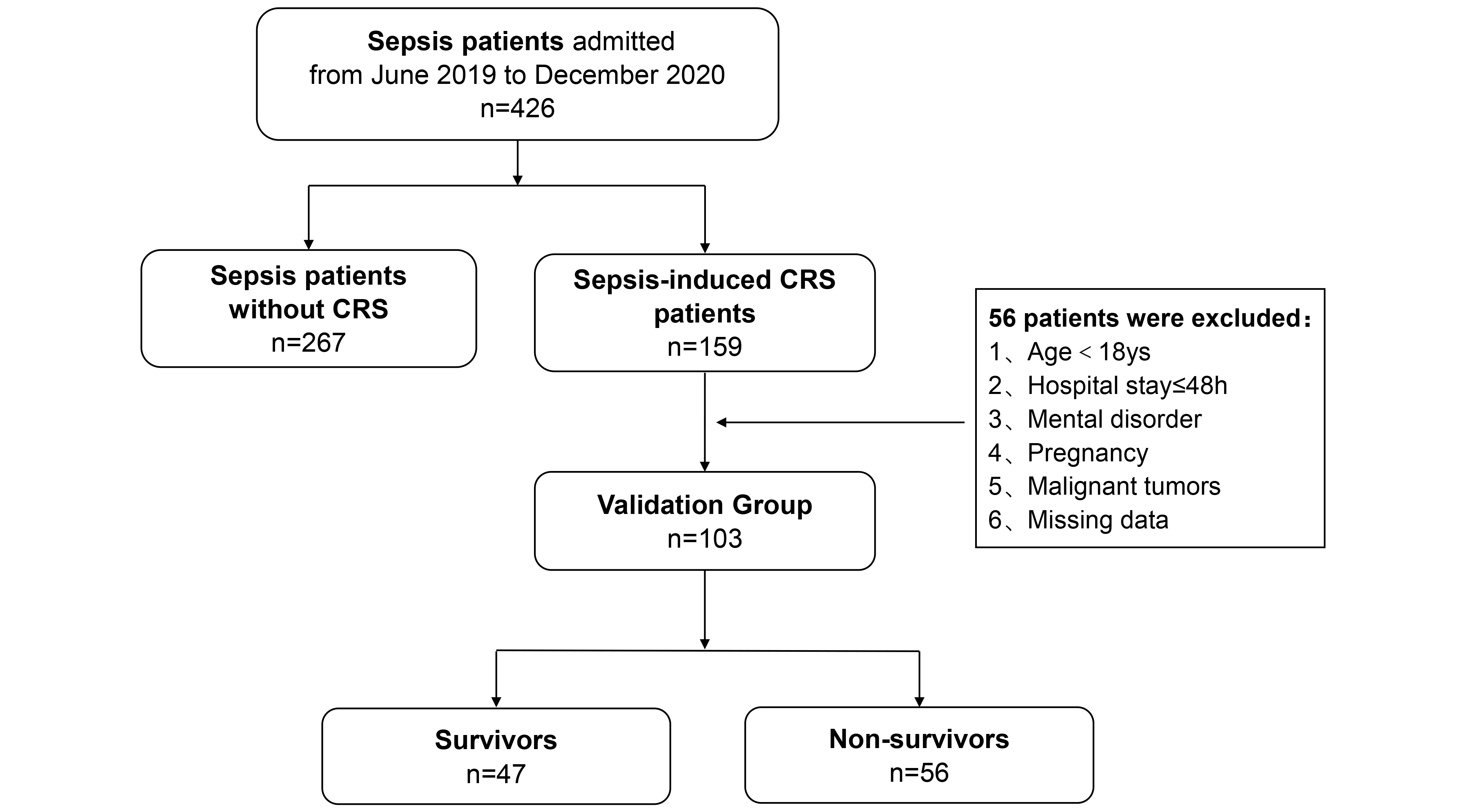

Supplement: Supplementary Figure 1 — Flow chart illustrating the patient enrollment in validation cohort. [file Image_1.JPEG]

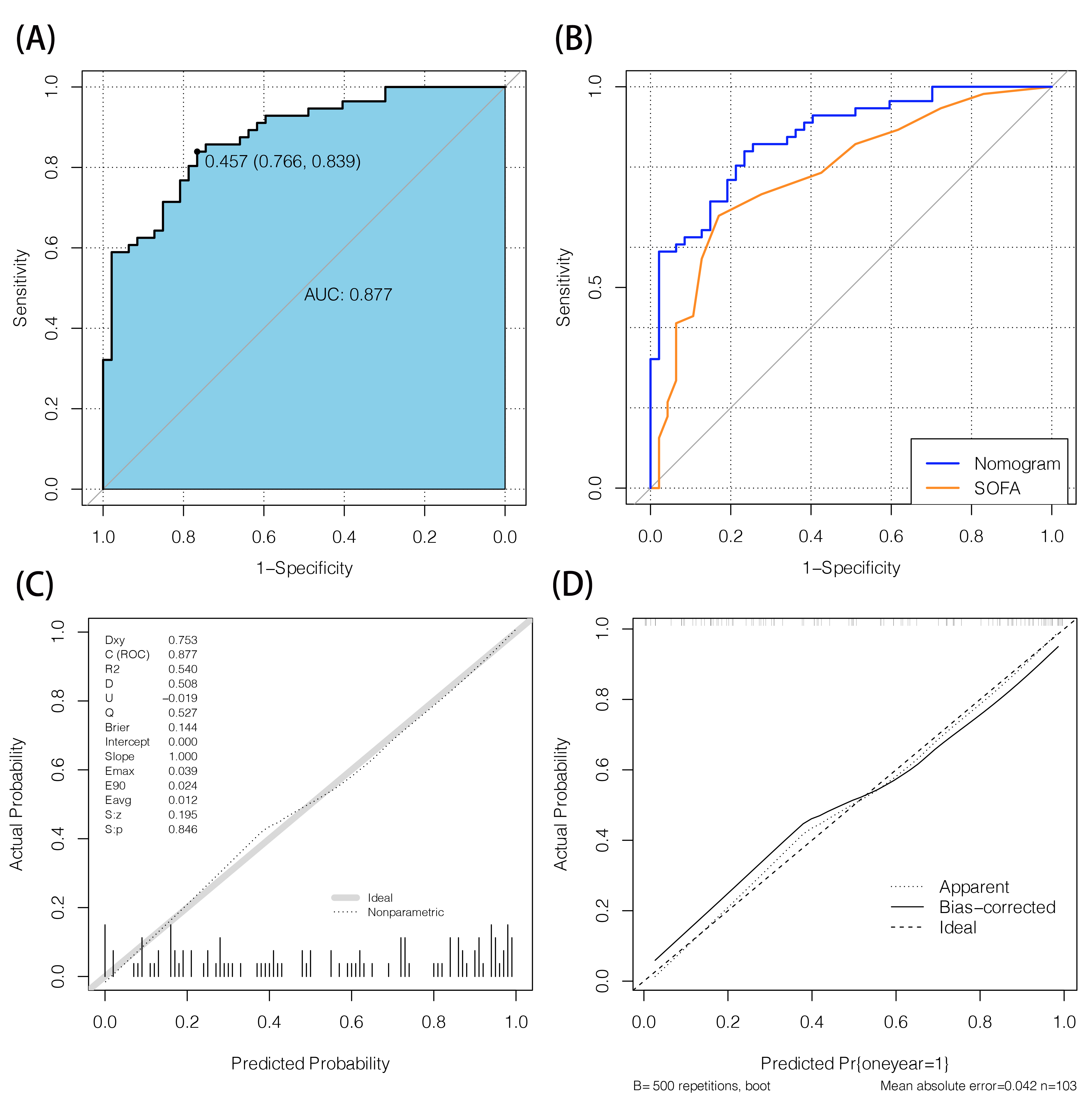

Supplement: Supplementary Figure 2 — The receiver operating characteristic (ROC) curve and calibration curve of nomogram in external validation. (A) The ROC curves of the nomogram; (B) comparison of AUC between the nomogram and SOFA; (C) the calibration curve of the nomogram; (D) comparison of the calibration curves between the ideal model, the nomogram and the bias-corrected model. [file Image_2.JPEG]

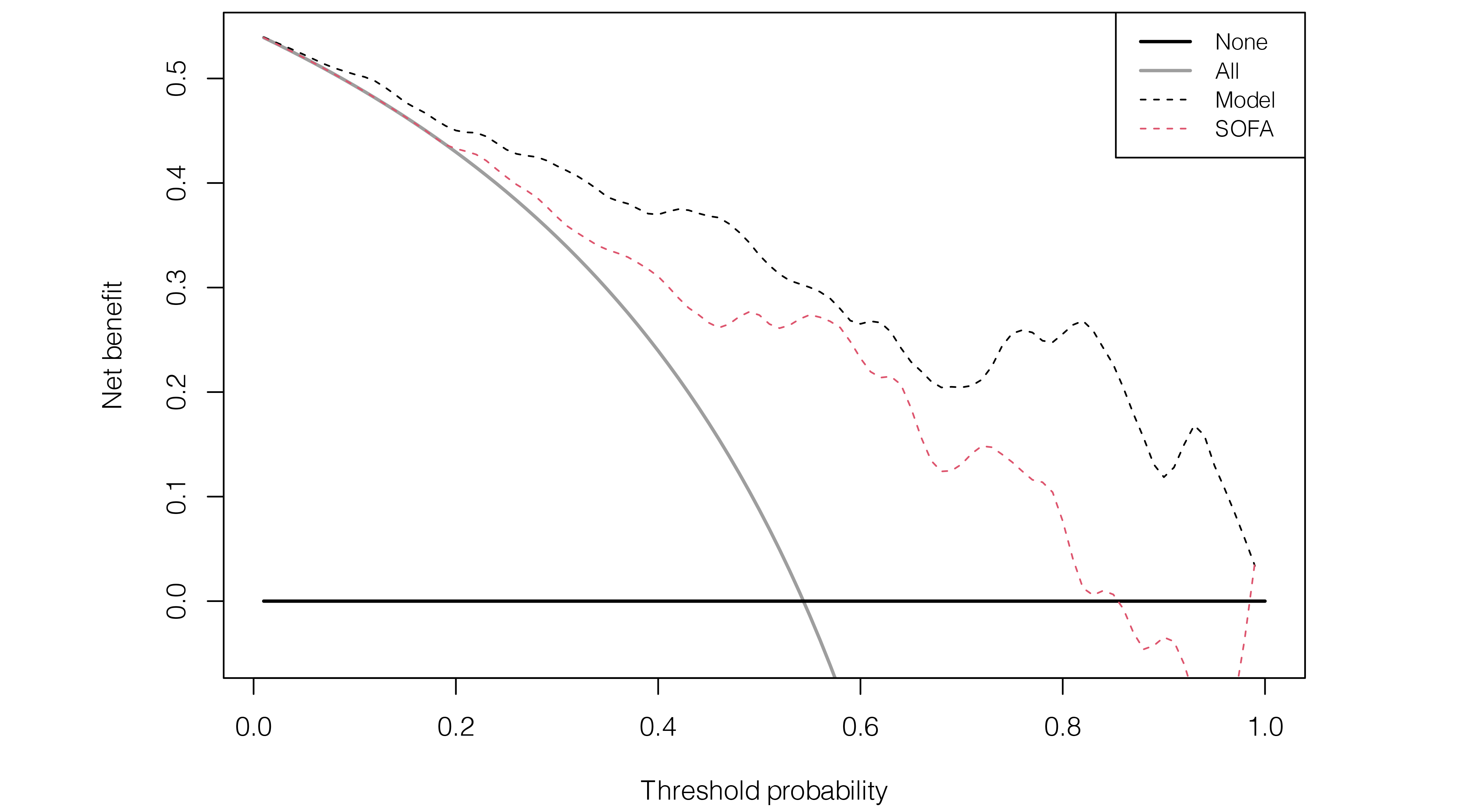

Supplement: Supplementary Figure 3 — The decision analysis curves of the nomogram and SOFA in external validation. [file Image_3.JPEG]

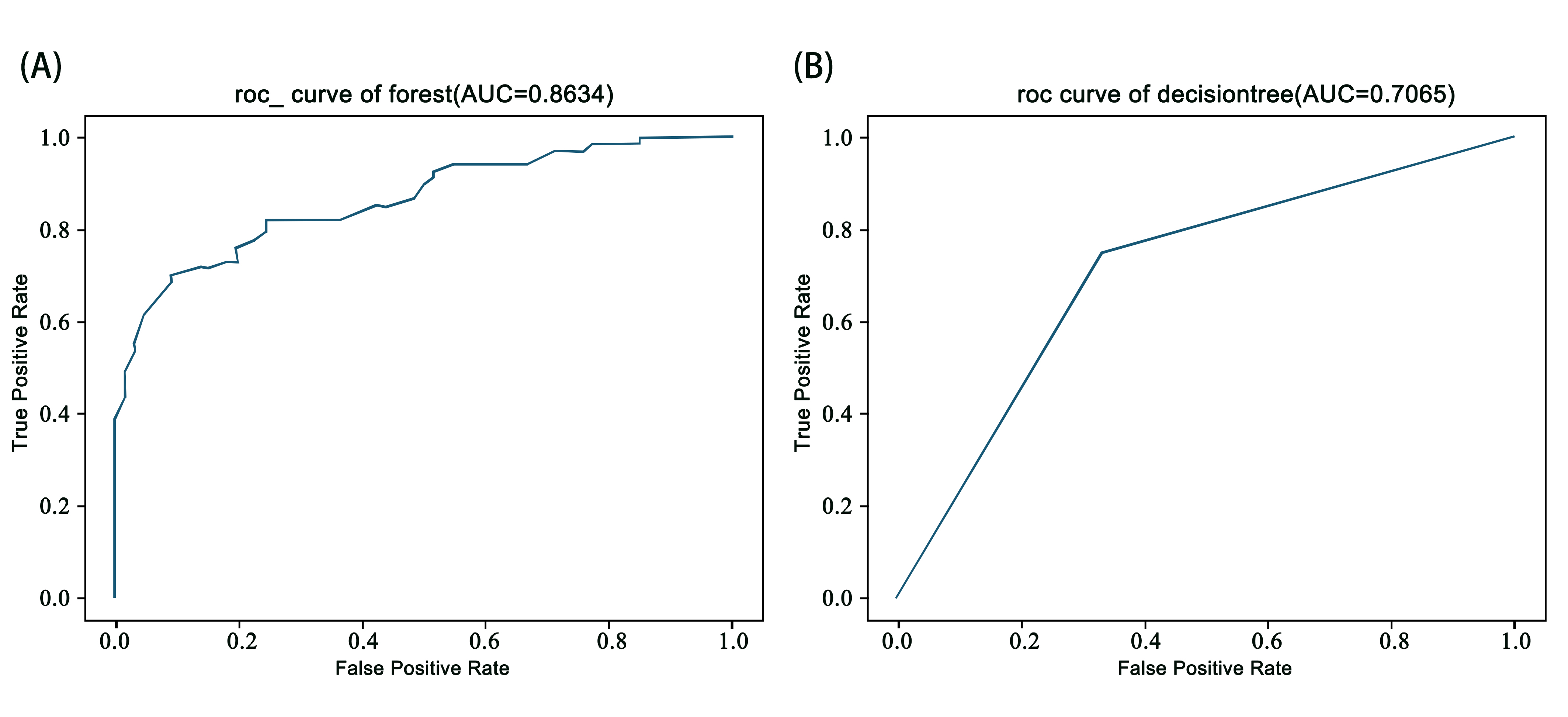

Supplement: Supplementary Figure 4 — The ROC curves of machine learning models in external validation. (A) ROC curves of Random Forest; (B) ROC curves of decision tree. [file Image_4.JPEG]
